# Estimated numbers of cases with HIV from 1990 to 2010 in Fars province by capture recapture method

**DOI:** 10.1186/1742-4690-9-S1-P87

**Published:** 2012-05-25

**Authors:** M Marzban, H Joulaee, P Kazeroni

**Affiliations:** 1 Epidemilogy at HIV Reseach Center, Shiraz University of Medical Science, Shiraz, Iran

## Background

HIV is potential risk in different countries of the world. According to UNAIDS, estimated HIV-infected people in Iran 4 times more than the cases have been recorded. Full details of the cases with HIV is one of the most essential tool for planning. The aim of the study is to estimate numbers of infected patients during the selected time.

## Method

All information contained in three sources hospital, voluntary counseling and testing center (VCT) and prison from 1990 to 2010 were used. Then by record linkage identify common cases finally log-linear methods were fit for eight models.

## Result

5167 cases entered to the study. The proportion of men 10 times women also most patients belong to the age group 15 to 44 years old. The main source of recorded cases is voluntary counseling and testing center by 3347 patients. As expected the lowest source is the common cases between prison and hospitals. annually average of 550 patients have been added. The lowest estimate belong to the common cases between prison and hospital but most of them are the presence of three sources. Estimated number of cases in the province are approximately 18914.

## Conclusion

Low coordination between the various components of the health system, along with social issue such as stigma and discrimination of HIV positive patients is the most important weakness in information systems. Therefore constitutional arrangements for providing high quality information is essential for HIV-infected cases and should be the priorities of health policy investment.

